# FOXP3‐regulated lncRNA NONHSAT136151 promotes colorectal cancer progression by disrupting QKI interaction with target mRNAs

**DOI:** 10.1111/jcmm.18068

**Published:** 2023-12-02

**Authors:** Handong Huang, Xiaoxiang Liang, Weizheng Wu, Tao Yuan, Zhengquan Chen, Lin Wang, Zhenyu Wu, Tao Zhang, Kai Yang, Kunming Wen

**Affiliations:** ^1^ Soochow University Medical College Suzhou Jiangsu China; ^2^ Department of General Surgery Affiliated Hospital of Zunyi Medical University Zunyi Guizhou China

**Keywords:** colorectal cancer, long non‐coding RNA, NONHSAT136151, QKI, RNA binding protein

## Abstract

The role of lncRNAs in the pathogenesis of cancer, including colorectal cancer (CRC), has repeatedly been demonstrated. However, very few lncRNAs have been well annotated functionally. Our study identified a novel lncRNA upregulated in CRC, NONHSAT136151, which was correlated with clinical progression. In functional assays, NONHSAT136151 significantly enhanced CRC cell proliferation, migration and invasion. Mechanistically, NONHSAT136151 interacted with RNA‐binding protein (RBP) QKI (Quaking) to interfere with QKI binding to target mRNAs and regulate their expression. As well, FOXP3 may be causally related to the dysregulation of NONHSAT136151 in CRC cells through its transcriptional activity. In conclusion, our findings identified a novel lncRNA regulated by FOXP3 participates in CRC progression through interacting with QKI, indicating a novel lncRNA‐RBP interaction mechanism is involved in CRC pathogenesis.

## 1 INTRODUCTION

According to the latest cancer statistics worldwide, colorectal cancer (CRC) is the third most common cancer and the second most prevalent cause of cancer‐related deaths.^1^ CRCs unrelenting high morbidity and poor prognosis have caused significant economic and psychological burden worldwide.^2^ Deepening the understanding of pathogenic mechanisms at the molecular level is imperative to identify the new specific biomarkers and effective targets of treatment in CRC. It has become increasingly apparent in the past decade that lncRNAs take part in the occurrence and progression of numerous diseases in humans,^3^, ^4^, ^5^, ^6^, ^7^, ^8^, ^9^ including malignant tumours.^7^, ^8^, ^9^ Accumulating studies have revealed that lncRNA expression is frequently dysregulated in CRC, which often leads to changes in various tumour phenotypes, such as proliferation, apoptosis and invasion.^10^ It follows that lncRNAs are potentially valuable biomarkers and therapeutic targets in CRC.

LncRNAs are reported to perform regulatory functions at almost all levels of gene expression through the lncRNA interactome, which refers to various cellular biomolecules including DNA, RNA, miRNA and proteins.^11^, ^12^ However, most scholars believe that lncRNAs function mainly by interacting with RNA‐binding proteins (RBPs).^13^, ^14^, ^15^ According to recent studies, the interaction of lncRNAs with RBPs also participates in CRC pathogenesis. For instance, binding of lncRNA OCC‐1 enhanced ubiquitination of HuR, suppressing the growth of CRC cells^16^; LINC0051 promoted proliferation of CRC in vitro by binding to EZH2 and enhancing its enrichment in the promoter region of IL‐24^17^; CYTOR bound to NCL and Sam68 as a scaffold and facilitated migration, invasion, proliferation and metastasis in CRC.^18^ Therefore, intensive research on the interaction between lncRNAs and RBPs is of great importance to thoroughly elaborating CRC pathogenesis.

The current study identifies NONHSAT136151 as an unannotated lncRNA involved in CRC progression. We also found that mechanistically, NONHSAT136151 exerted a pro‐oncogenic function by directly interacting with QKI (Quaking), a crucial RBP that is considered a tumour suppressor.

## 2 MATERIALS AND METHODS

### 2.1 Patient and clinical samples

In this study, 61 pairs of patient specimens, including colorectal carcinoma tumour tissues and paired adjacent noncancerous tissue (ANT), were collected from CRC patients undergoing radical resection at the Affiliated Hospital of Zunyi Medical University. The inclusion criteria were as follows: (a) histopathological confirmation from two experienced pathologists; (b) radical resection of CRC; (c) clinical records available. The exclusion criteria were: (a) preoperative antitumor treatments, such as chemotherapy, radiation and targeted therapy; (b) inflammatory bowel diseases, hereditary non‐polyposis CRCs or familial adenomatous polyposis; (c) other primary malignancies simultaneously. All tissue samples were snap‐frozen immediately following resection and stored at −80°C until subsequent use. In all cases, two pathologists with extensive experience confirmed the CRC diagnosis on histology. An ethics approval was obtained from the Medical Ethical Committee of Zunyi Medical University prior to the collection of tissue samples. All participating patients or their families provided formal consent.

### 2.2 Microarray analysis

Total RNA was extracted from paired CRC tumour and ANT samples. Clariom D human arrays (Affymetrix, Inc.) were utilized to screen the global profiling of human lncRNAs. Differentially expressed lncRNAs were defined as fold change ≥2.0 and *p*‐value ≤ 0.05. The chip assays and data analysis were performed by Beijing Cnkingbio Company.

Fluorescent labelled NONHSAT136151 was hybridised with the human proteome microarray (HuProt™ 20K; CDI Laboratories, Inc.) for NONHSAT136151‐binding protein detection. The assays and data analysis were performed by Shanghai Wayen Biotechnologies Inc., as previously described.^19^

### 2.3 Cell culture and transfection

Colorectal cancer cell lines, SW480 (cat. no. SCSP‐5033), SW620 (cat. no. TCHu101), Caco‐2 (cat. no. SCSP‐5027), HCT 116 (cat. no. TCHu 99) and HT‐29 (cat. no. SCSP‐5032), were purchased from the Cell Bank of the Chinese Academy of Sciences (Shanghai, China). Normal human colon cell FHC (cat. no. CRL‐1831) was purchased from the American Type Culture Collection (ATCC). DNA fingerprinting and mycoplasma contamination were performed on the cell lines. Cell lines were cultured in RPMI 1640 (HyClone, Cytiva) with 10% fetal bovine serum (FBS; Gibco, Inc.). SW480 and SW620 cells were kept at 37°C in an air incubator, and Caco‐2, HCT 116, HT‐29 and FHC cells were incubated in a humidified incubator at 37°C, with 95% air/5% CO_2_.

Small interfering RNAs (siRNAs) and corresponding negative control siRNA scramble (si‐NC) used in the study were purchased from Anhui General Biosystems, Inc. The siRNA sequences can be found in Table S1. CRC cells were cultured until 70–80% confluence and then treated with siRNAs using Lipofectamine 3000 Transfection Reagent (5 μL; cat. no. L3000075; Invitrogen, Thermo Fisher Scientific, Inc.). Transfected cells were collected for subsequent experiments after 48 hours incubation. RT‐qPCR or western blot was used to determine the knockdown efficiency after siRNA transfecting.

### 2.4 RNA extraction and quantitative reverse transcription poly‐merase chain reaction (RT‐qPCR)

Total RNA of cells and cryogenically ground tissue was extracted by RNAiso Plus (Takara Bio, Inc.). RNA reverse transcription was carried out under a conventional reaction condition (37°C for 15 min and 85°C for 5 sec) with a PrimeScriptTM RT Reagent Kit (cat. no. RR037A; Takara Bio, Inc.). Real‐time PCR was conducted with SYBR Premix Ex TaqTM II (cat. no. RR820A; Takara Bio, Inc.). The PCR program was as follow: Initial denaturation was carried out at 95°C for 30 s followed by 40 cycles of amplification at 95°C for 5 s and 60°C for 30 s, and melt curve analysis at 95°C for 15 s, 60°C for 30 s and 95°C for 15 s. Conventional $2^{-∆∆C_{q}}$ method was utilized to analyse the relative expression of RNAs.^20^ The sequences of primers can be found in Table S1.

### 2.5 Establishment of stable cell lines

The lentiviral vector pHBLV‐U6‐ZsGreen‐Puro containing the NONHSAT13151 short hairpin RNA (shNONHSAT136151) and shRNA scramble (shNC) was provided by Shanghai Hanbio Biotechnology Co., Ltd. The shNONHSAT136151 sequence was 5′‐UAGGCAUGGUUAGCGUGGUCGGAAU‐3′, and the shNC sequence was 5′‐TTCTTACACAATAGCCAGG‐3′. SW480 were treated with lentiviral particles at 20 MOI for 48 h at 37°C. Subsequently, puromycin (10 μg/mL; Gibco, Inc.) was utilized to select stably transfected cells for 4 weeks. NONHSAT136151 expression in SW480 cells was detected by RT‐qPCR.

### 2.6 Wound healing assay

Treated HCT 116 and SW480 were inoculated into 6‐well plates and cultured to 90% confluence. Then, a wound was scratched across well bottom with a 10 μL pipette tip. Cells were subsequently incubated in medium without FBS for 48 h. The scratches were photographed to measure the wound healing area at 0 and 48 h (Leica DMil LED, Leica Biosystems Co., Ltd.), and wound closure percentage was calculated.

### 2.7 Colony formation assay

CRC cells were re‐plated into 12‐well plates (1000 cells per well) and further cultured for 7 days. Then, the cells were fixed with 4% paraformaldehyde for 30 min and stained with Giemsa for 15 min. Finally, photographs and counts were taken of colonies containing at least 50 cells.

### 2.8 Transwell invasion assays

Transwell assays were used to test invasion capacity of cells with BD BioCoat Matrigel Invasion Chamber (BD Biosciences, Inc.; pore size, 8 μm). Cells (5 × 10^4^) were seeded in the upper chamber and incubated in medium without FBS. Medium with FBS was added into the lower chamber. After 24 h, cells that had passed through Matrigel were treated for 1 h using 4% paraformaldehyde in the bottom of chamber, whereas cells retained in the upper chamber were eliminated. After washed with PBS, fixed cells were stained using 1% crystal violet for 10 min. Cells were counted and photographed through a microscope (Leica DMil LED, Leica Biosystems Co., Ltd.).

### 2.9 RNA fluorescent in situ hybridization (FISH)

Briefly, SW480 and HCT 116 cells were fixed using 4% paraformaldehyde for 20 min at 4°C. Subsequently, the cells were cultured in hybridization buffer containing FITC‐labelled NONHSAT136151 probes (Wuhan Servicebio Technology Co., Ltd.) overnight. Next, DAPI (RiboBio, Inc.) staining of the cells was performed in dark for 10 min. FISH signals were analysed with a fluorescence microscope (Nikon E800, Nikon Corporation).

### 2.10 Subcellular fractionation analysis

According to the manufacturer's instructions, cytoplasmic RNA was isolated from nuclear RNA using a Cytoplasmic & Nuclear RNA Purification Kit (Norgen, Norgen Biotek Corp.). SW480 and HCT 116 cells were lysed with the lysis buffer J, followed by centrifugation. Subsequently, cytoplasmic RNA was isolated from the supernatant, while the precipitate was collected to extract the nuclear RNA. RNAs isolated from cytoplasm and nuclear were examined by RT‐qPCR. A cytoplasmic control was GAPDH, while a nuclear control was NEAT1.

### 2.11 Tumorigenicity assay in vivo

Tumorigenicity assay was performed on 5–6–week‐old male BALB/c mice (*n* = 14) provided by Hua Fukang Biological Technology Co., Ltd. 2 × 10^6^ SW480 cells stably transfected with shNONHSAT136151 or shNC were inoculated subcutaneously into the right flank of mice in two groups (7 mice each), respectively. The length and width of tumour were measured on alternate days and the formula (length × width^2^)/2 was used to calculate tumour volume. After 30 days, mice euthanasia was performed using 1% pentobarbital sodium injected intraperitoneally and cervical dislocation. Subsequently, tumours were completely excised and then stored in −80°C. The humane endpoints in our animal experiment included: (a) Tumour burden multiplied by 10% of body weight; (b) starting body weight lost more than 20%; (c) tumour became ulcerated or appeared to be an infection; (d) complete loss of appetite >24 h or food intake less than 50% of normal >72 h; (e) weakness (inability to eat or drink). Neither mice died nor did animals reach the humane endpoints in this experiment.

### 2.12 RNA pull‐down assay

NONHSAT136151 and its antisense transcripts were provided commercially (GENEWIZ Biotechnology Co., Ltd.) and labelled with biotin using Pierce RNA 3′ End Desthiobiotinylation Kit (cat. no. 20163; Thermo Fisher Scientific, Inc.). lncRNA pull‐down assays were carried out with Pierce Magnetic RNA‐Protein Pull‐Down Kit (cat. no. 20164; Thermo Fisher Scientific, Inc.). Labelled RNA that was captured by Streptavidin Magnetic Beads (cat. no. 88816; Thermo Fisher Scientific, Inc.) were incubated in cell lysates at 4 °C for 1 h with rotation. After RNA‐protein complexes were washed and eluted, separating co‐precipitated proteins by SDS‐PAGE was performed and visualized with Fast Silver Stain Kit (cat. no. P0017S; Beyotime Biotechnology, Co., Ltd.). Finally, the protein samples were examined by western blot or mass spectrometry.

### 2.13 Western blot analysis

Whole cell lysates were generated conventionally using RIPA lysis buffer (cat. no. P0013B, Beyotime Biotechnology, Co., Ltd.). Next, 40 μg protein samples were separated by 12.5% SDS‐PAGE gels and blotted to polyvinylidene difluoride membranes (PVDF; Millipore; Merck KGaA). After blocked with skim milk (5%), PVDF membranes were incubated with primary antibodies at 4°C overnight and with secondary antibodies at room temperature for 1 h. Protein bands were visualized with Super ECL Plus (Applygen Technologies, Inc.) and scanned with a camera (Leica ICC50W, Leica Biosystems Co., Ltd.). Finally, the Image J software (version 1.51j8; National Institutes of Health) was utilized to conduct the densitometric analysis. The antibodies used in this study are listed in Table S3.

### 2.14 Protein immunofluorescence‐RNA FISH double labeling (IF/FISH)

FISH assay was performed on fixed CRC cells as aforementioned. Subsequently, the slides were incubated with a primary antibody for QKI (1:1000; cat. no. HPA019123; Sigma‐Aldrich) overnight at 4°C. The next day, cells were washed with PBS three times and then incubated with a goat anti‐rabbit IgG H&L secondary antibody (Alexa Fluor® 594) (1:200; cat. no. ab150080; Abcam) for 1 h. Subsequently, slides were washed three times with PBS and then counterstained with DAPI (RiboBio, Inc.) for nuclei staining. Finally, anti‐fluorescence quencher was used to seal the sections. Fluorescent images were acquired using a microscope (Leica DMil LED, Leica Biosystems Co., Ltd.) equipped with a digital colour camera (Leica DFC450C, Leica Biosystems Co., Ltd.).

### 2.15 RNA immunoprecipitation (RIP) assay

RIP assay was conducted using the Imprint RNA Immunoprecipitation Kit (Sigma‐Aldrich Co., Ltd.). Briefly, SW480 cells treated with siRNA were lysed with RIP lysis buffer. Anti‐QKI antibody (cat. no. HPA019123; Sigma‐Aldrich Co., Ltd.) or IgG from rabbit serum were pre‐bound to Protein A Magnetic Beads and then incubated with cell lysate at 4°C overnight with rotation. The magnetic bead‐antibody QKI complex was washed and resuspended with RIP wash buffer. RNA was recovered using TRI Reagent (cat. no. T9424; Sigma‐Aldrich Co., Ltd.) and dissolved in RNAse‐free water. Subsequently, PrimeViewTM Human Gene Expression Array (Affymetrix Inc.) was utilised to analyse the RNA samples. Gene chip assay and data analysis were commissioned to Beijing Cnkingbio Company.

### 2.16 Bioinformatic databases

Coding potential score of NONHSAT136151 was analysed using the Coding Potential Calculator 2 (CPC2) (http://cpc2.cbi.pku.edu.cn.) and Coding Potential Assessment Tool (CPAT) (http://lilab.research.bcm.edu/). Gene differential expression analysis was carried out with GO enrichment (http://www.geneontology.org/) and KEGG enrichment (http://www.genome.jp/kegg/). The putative transcription factors were predicted and filtered using the following online tools: PROMO (http://alggen.lsi.upc.es/cgi‐bin/promo_v3/promo/promoinit.cgi?dirDB=TF_8.3), JASPAR (https://jaspar.genereg.net/) and Gene Expression Profiling Interactive Analysis (GEPIA) (http://gepia.cancer‐pku.cn/).

### 2.17 Chromatin immunoprecipitation (ChIP)

Chromatin immunoprecipitation was performed using a Pierce Magnetic ChIP kit (Thermo Fisher Scientific, Inc.). In brief, paraformaldehyde‐fixed cells were lysed, and then the chromatin was sonicated to fragments with the length of 200–700 bases. The chromatin samples were immunoprecipitated with anti‐FOXP3 antibody (cat. no. 22228‐1‐AP; Proteintech) or equal normal IgG at 4°C with rotation overnight. Subsequently, ChIP‐Grade Protein A/G Magnetic Beads were incubated with protein/chromatin complexes at 4°C for 2 h. After elution and purification, the DNA fragments were examined by qPCR. Primers used to amplify NONHSAT136151 promoter‐specific fragments are listed in Table S2.

### 2.18 Statistical analysis

Data in this study are presented as mean ± standard deviation (SD). GraphPad Prism 6.0 (GraphPad Software, Inc.) or SPSS 19.0 (SPSS, Inc.) were utilized for statistical analysis. NONHSAT136151 differential expression in CRC tissue and ANT was analysed using Wilcoxon matched pairs signed rank test. Other differential analysis between two groups were carried out by the Student's *t*‐test or Mann–Whitney *U* test. The association between NONHSAT136151 expression and clinicopathological features was analysed by Chi‐square or Fisher's exact test. The correlation between expression of NONHSAT136151 and QKI in CRC tissues was analysed by Spearman's rank correlation test. A *p*‐value < 0.05 was considered statistically significant.

## 3 RESULTS

### 3.1 Identification of lncRNAs with differential expression between CRC tissue and ANT

**FIGURE 1:**
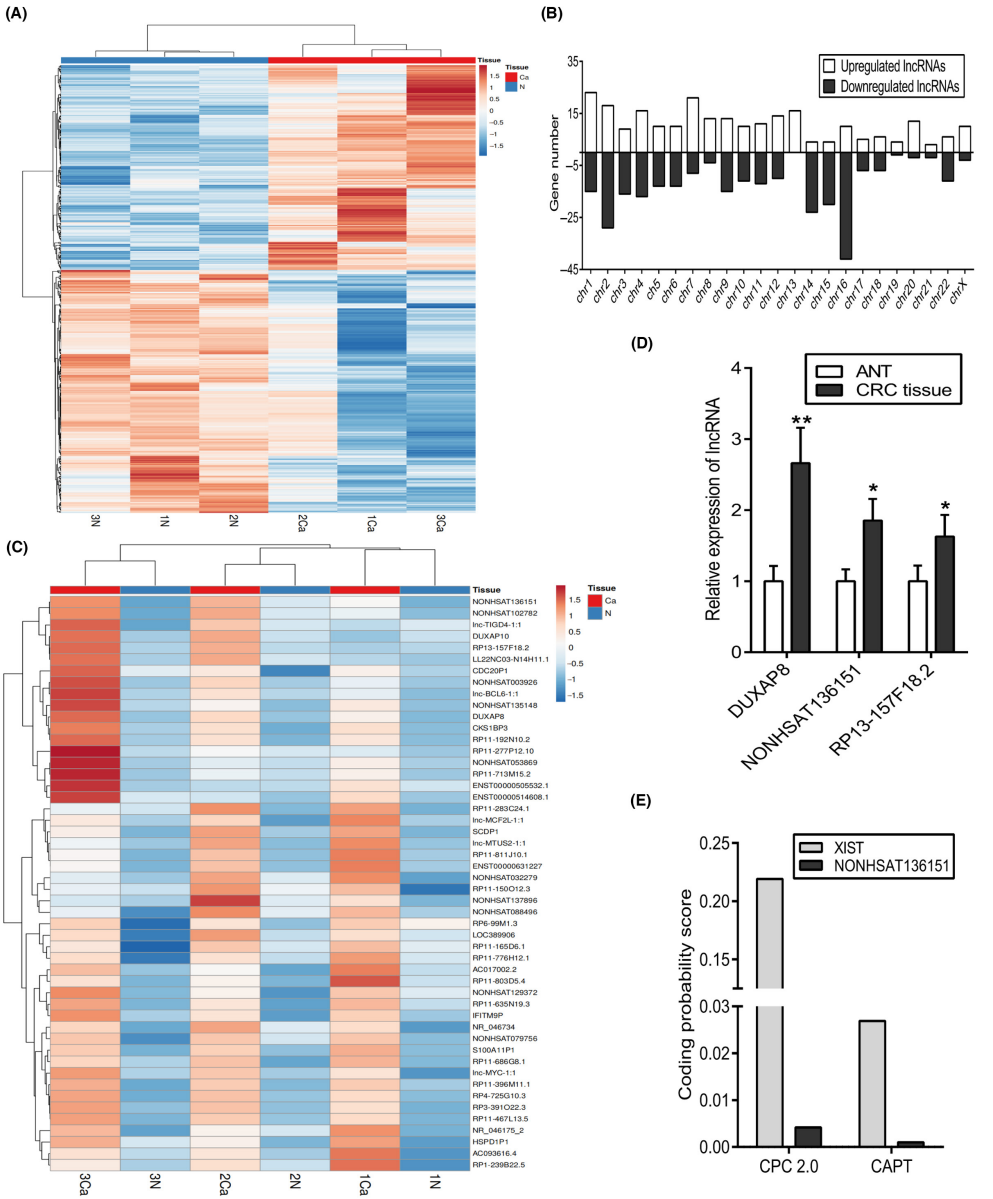
Identification of CRC‐related lncRNAs. (A) Heatmap represented the differential expression of lncRNAs in three CRC samples compared to corresponding ANTs (fold change >2; p value < 0.05). (B) Distribution of differentially expressed lncRNAs on human chromosomes. (C) Top 50 upregulated lncRNAs according to the data of gene chip. (D) Relative expression of DUXAP8, NONHSAT136151 and RP13‐157F18.2 was detected in the three paired samples by RT‐qPCR (n = 3). (E) CPC 2.0 and CAPT were used to compare the coding potential of NONHSAT136151 and control lncRNA XIST. Results were shown as the mean ± standard deviation based on 3 independent experiments, *p < 0.05, **p < 0.01 vs. the ANT group. ANT, adjacent noncancerous tissue; CAPT, Coding Potential Assessment Tool; CPC, Coding Potential Calculator; CRC, colorectal cancer; XIST, X‐inactive specific transcript.

Firstly, we examined the lncRNA profiles differentially expressed in paired CRC tumour and paracancer tissues (*n* = 3) using gene microarray. Based on fold change >2.0 and *p* < 0.05, 537 lncRNAs were identified as differentially expressed genes in CRC (246 with high expression and 291 with low expression) (Figure 1A). The origins of these lncRNAs were widely distributed in all human chromosomes (Figure 1B). The top 50 upregulated genes are presented in Figure 1C. The top 3 upregulated lncRNAs (DUXAP8, NONHSAT136151 and RP13‐157F18.2) had fold changes greater than 6.0. RT‐qPCR analysis of the three pairs of samples indicates CRC tissue expresses the three lncRNAs at higher levels than ANT (Figure 1D). One of the most upregulated lncRNA, NONHSAT136151, currently has no detailed functional annotation. According to the NONCODE and LNCipedia databases, NONHSAT136151 is an intergenic lncRNA located on chromosome X that is 210 nucleotides in length. Coding potential analysis showed that both CPC 2.0 and CPAT had a lower coding probability than XIST, a well‐known lncRNA (Figure 1E). Therefore, NONHSAT136151 can be potentially regarded as a CRC‐related lncRNA.

### 3.2 NONHSAT136151 was significantly highly expressed in CRC tissue and clinically associated with CRC progression

**FIGURE 2:**
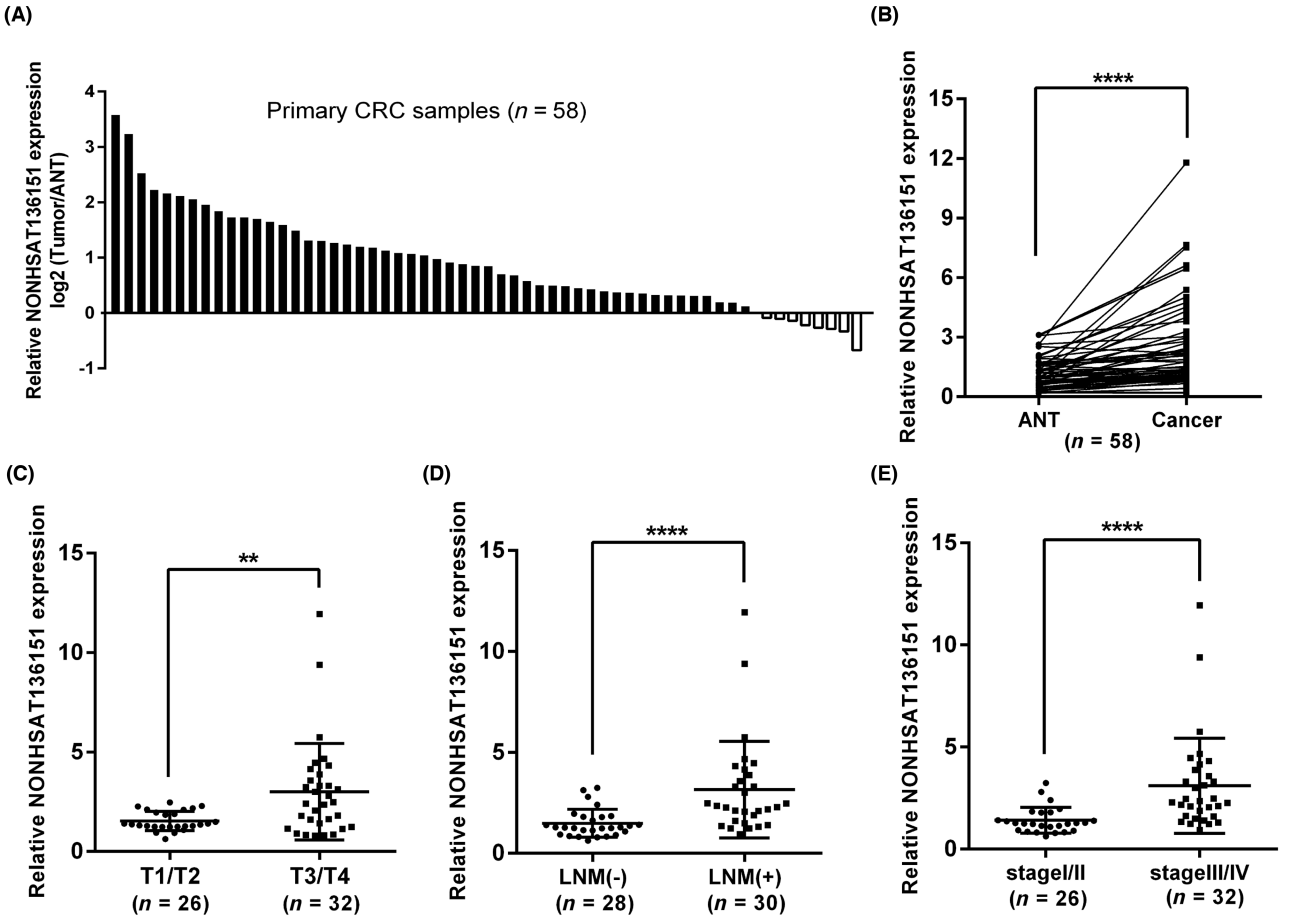
The expression of NONHSAT136151 in CRC tissues and the association between NONHSAT136151 expression and CRC progression. (A) and (B) RT‐qPCR was used to analyse the expression of NONHSAT136151 in CRC tissues and corresponding ANTs (*n* = 58). ****p < 0.0001 vs. the ANT group. (C), (D) and (E) Relative NONHSAT136151 expression was measured by RT‐qPCR in CRC tissues from patients of different tumour infiltration, lymph node metastasis (LNM) and TNM stages (*n* = 58). **p < 0.01, ****p < 0.0001. ANT, adjacent noncancerous tissue; CRC, colorectal cancer; LNM, lymph node metastasis.

To investigate NONHSAT136151 expression in CRC and the association between the two, we collected another 58 pairs of CRC and ANT specimens together with the corresponding clinicopathological data of the patients. Like microarray data, RT‐qPCR data also showed that NONHSAT136151 was significantly over‐expressed in tumours compared to ANTs (Figure 2A,B). Subsequently, the clinicopathological features of the 58 patients including gender, age, preoperative CEA, tumour size, differentiation degree, infiltration, lymph node metastasis (LNM), TNM staging and distant metastasis were analysed after being grouped by NONHST136151 expression levels. Those with a relative expression value higher than or equal to the median (*n* = 29) were classified as high expression; those expressing lower levels than median (*n* = 29) were considered as low expression. Analysis of statistical data demonstrated a significant association of upregulated NONHSAT136151 expression with LNM, tumour infiltration and TNM staging rather than other clinicopathological characteristics (Table 1). As expected, NONHSAT136151 was expressed significantly higher in cases with deeply infiltrated tumours (T3 and T4) compared to that with superficially infiltrated tumours (T1 and T2) (Figure 2C); higher expression of NONHSAT136151 was detected in CRC samples with LNM than in those without LNM (Figure 2D). Analogously, CRC cases in stages III and IV expressed higher levels of NONHSAT136151 than stage I and II cases (Figure 2E). In conclusion, the aforementioned findings demonstrate that NONHSAT136151 exhibits elevated expression levels in CRC tissue in comparison to ANT, and this heightened expression is significantly associated with the advancement of CRC.

**TABLE 1:** Correlation between NONHSAT136151 expression and clinicopathologic features (*n* = 58).

Clinicopathologic features	All patients	Expression of NONHSAT136151		*p*‐Value
		Low (*n* = 29)	High (*n* = 29)	
Gender				NS
Male	32(55.2%)	15	17	
Female	26(44.8%)	14	12	
Age, years				NS
<60	30(51.7%)	14	16	
≥60	28(48.3%)	15	13	
Preoperative CEA, μg/L				NS
<5	30(51.7%)	15	15	
≥5	28(48.3%)	14	14	
Tumour size, cm				NS
<5	32(55.2%)	17	15	
≥5	26(44.8%)	12	14	
Histological differentiation				NS
Well	9(15.5%)	5	4	
Moderate	41(70.7%)	19	22	
Poor	8(13.8%)	5	3	
Tumour infiltration				0.035
T1 + T2	25(43.1%)	17	9	
T3 + T4	33(56.9%)	12	20	
Lymph node metastasis				<0.001
Yes	30(51.7%)	8	22	
No	28(48.3%)	21	7	
Distant metastasis				NS
Yes	4(6.9%)	1	3	
No	54(93.1%)	28	26	
TNM stage				<0.001
I + II	26(44.8%)	20	6	
III + IV	32(55.2%)	9	23	

*Note*: Metastatic statuses of 4 patients at stage IV: distant metastasis (4 with liver metastasis at M1a stage), lymph node metastasis (2 with N0, 1 with N1c and 1 with N2b). CRC patients were divided into low and high group according to median expression of NONHSAT136151. Chi‐square or Fisher's Exact test was used to evaluate the differences among variables.

Abbreviations: NS, not significant between different groups; TNM stage, tumour‐node‐metastasis stage.

### 3.3 Highly expressed NONHSAT136151 was associated with CRC cell proliferation, migration and invasion

**FIGURE 3:**
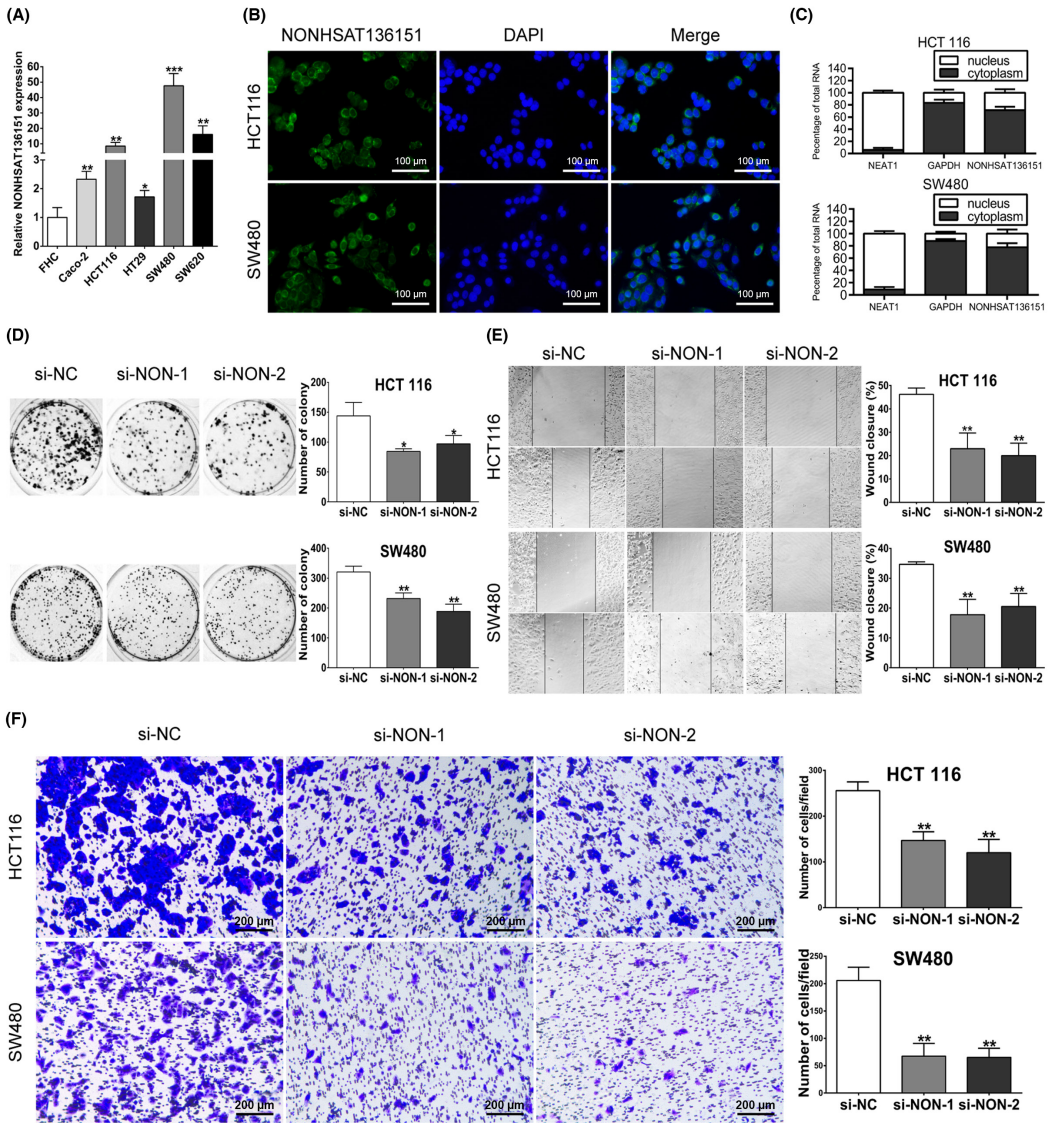
Expression characteristics and regulatory function of NONHSAT136151 in CRC cells. (A) The NONHSAT136151 expression in Caco‐2, HCT 116, HT 29, SW480 and SW620 cells normalized to the expression in FHC cells. *p < 0.05, **p < 0.01 and ***p < 0.001 vs. the FHC group. (B) The subcellular location of NONHSAT136151 in HCT 116 and SW480 cells presented by fluorescent in situ hybridization. (C) Subcellular fractionation analysis of NONHSAT136151 in HCT 116 and SW480 cells. (D) Colony formation assays were preformed to examine the effect of NONHSAT136151 silencing on proliferation of CRC cells. Magnification, ×200. (E) The migration of HCT 116 and SW480 cells after NONHSAT136151 silencing was examined by scratch wound healing assays. Magnification, ×100. (F) Transwell invasion assays were used to test the invasion suppression of CRC cells after NONHSAT136151 silencing. Results were exhibited as the mean ± standard deviation on the basis of 3 independent experiments, *p < 0.05, **p < 0.01 vs. the si‐NC group. CRC, colorectal cancer; DAPI, 4′,6‐diamidino‐2‐phenylindole; FHC, Fetal Human Colon cell, a normal human colon cell line; NC, negative control.

Subsequently, we investigated NONHSAT136151 expression level in several human CRC cells (HCT 116, SW480, Caco‐2, HT‐29 and SW620) and one human colon epithelial cell (FHC) using RT‐qPCR. Compared to FHC, all five cell lines of CRC expressed higher levels of NONHSAT136151 (Figure 3A). Furthermore, we performed RNA fluorescent in situ hybridization in HCT 116 as well as SW480. According to the results, the main distribution of NONHSAT136151 was in the cytoplasm (Figure 3B). Additionally, subcellular fractionation analysis showed a similar result that a high percentage of NONHSAT136151 was found within the cytoplasm (Figure 3C). Therefore, NONHSAT136151 is a lncRNA mainly distributed in the cytoplasm.

As NONHSAT136151 was highly expressed in CRC, we hypothesized it might be associated with colorectal carcinogenesis. Therefore, we silenced NONHSAT136151 expression of CRC cell lines and carried out colony formation assays. The results showed a significantly reduced colony numbers in NONHSAT136151‐silenced group, which indicated that NONHSAT136151 facilitated the cell proliferation of CRC (Figure 3D). Subsequently, scratch wound healing assays showed that knockdown of NONHSAT136151 shortened the migration distance of tumour cells in serum‐free medium (Figure 3E). Similarly, we found that knockdown of NONHSAT136151 impaired the invasion of CRC cells in transwell invasion assays (Figure 3F). Generally, these experiments revealed that lowering NONHSAT136151 expression in CRC cells was sufficient to suppress the malignant phenotype by decreasing proliferation, invasion and migration, which potentially indicates the promotive effect of NONHSAT136151 on CRC progression.

### 3.4 Knockdown of NONHSAT136151 impaired CRC tumorigenic capacity in vivo

**FIGURE 4:**
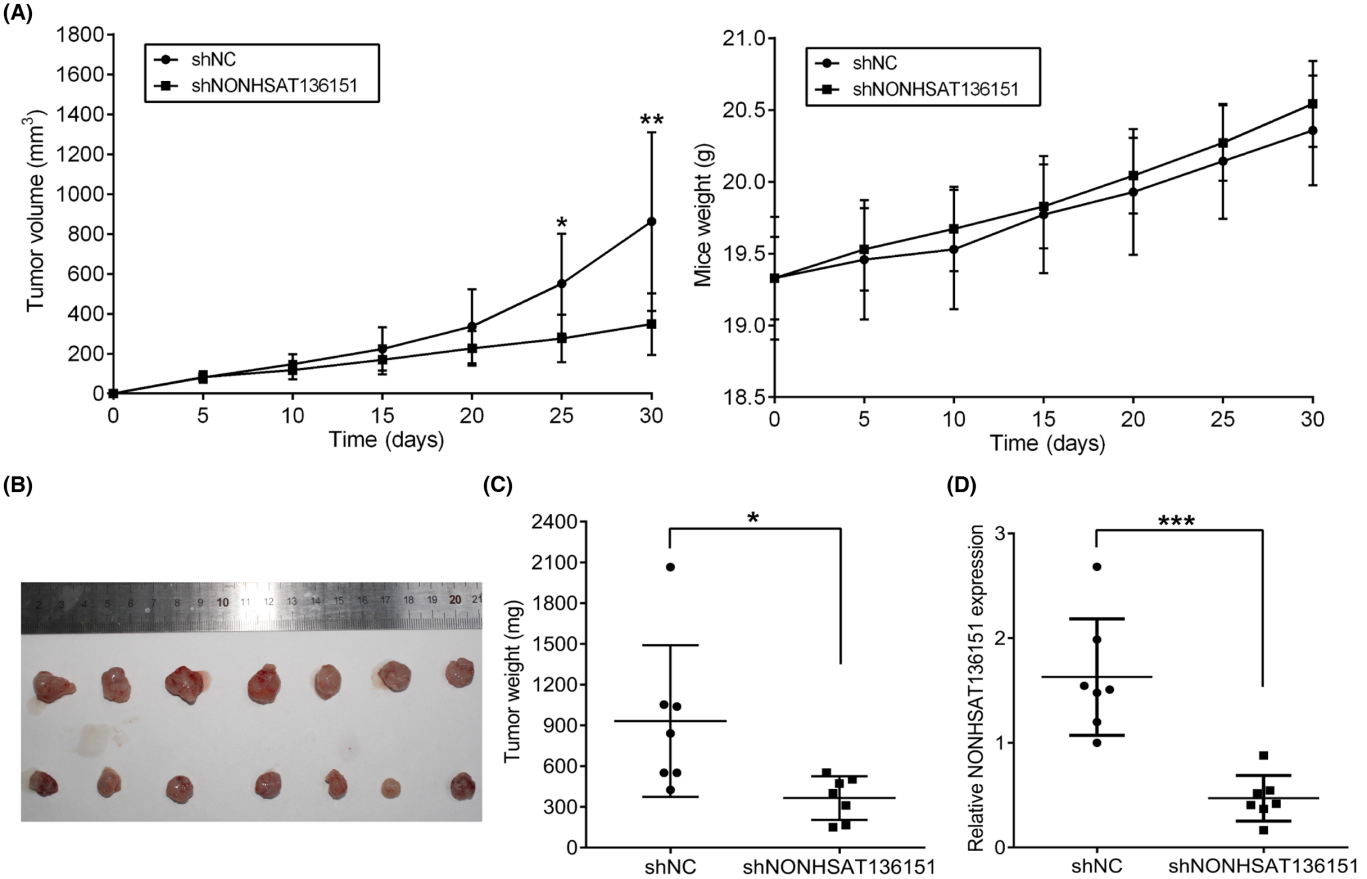
NONHSAT136151 silencing impaired the growth of CRC in vivo. (A) Tumorigenicity assays were performed in nude mice. Mean tumour volumes and mice weights of each group xenografts (NONHSAT136151 silencing group and shNC group) were measured every 5 days after SW480 cells injection (*n* = 7). (B, C) Tumour weights and volumes of xenografts were decreased after NONHSAT136151 knockdown (*n* = 7). (D) RT‐qPCR was used to detect the expression of NONHSAT136151 in SW480/shNONHSAT136151‐xenografted tumours and the control ones (*n* = 7). The measurement data are expressed as mean ± standard deviation. *p < 0.05, **p < 0.01 and ***p < 0.001 vs. the shNC group. CRC, colorectal cancer; NC, negative control.

To determine whether NONHSAT136151 function is responsible for CRC growth, we injected SW480 cells treated by shNONHSAT136151 and shNC into BALB/c mice subcutaneously. A growth curve was plotted based on subcutaneous tumour size and mouse weight measurements over time. The results indicated that NONHSAT136151 knockdown resulted in significant growth inhibition of xenograft tumours without affecting mice's weight gain (Figure 4A). Excised subcutaneous tumours were measured for size and weight, and the data showed that SW480/shNONHSAT136151‐xenograft tumours were smaller than SW480/shNC‐xenograft tumours (Figure 4B,C). As expected, SW480/shNONHSAT136151‐xenograft tumours had lower expression of NONHSAT136151 than SW480/shNC‐xenograft tumours (Figure 4D). Therefore, stable downregulation of NONHSAT136151 was sufficient to continuously suppress the tumorigenicity of CRC cells.

### 3.5 NONHSAT136151 bound to the RBP QKI in CRC cells

**FIGURE 5:**
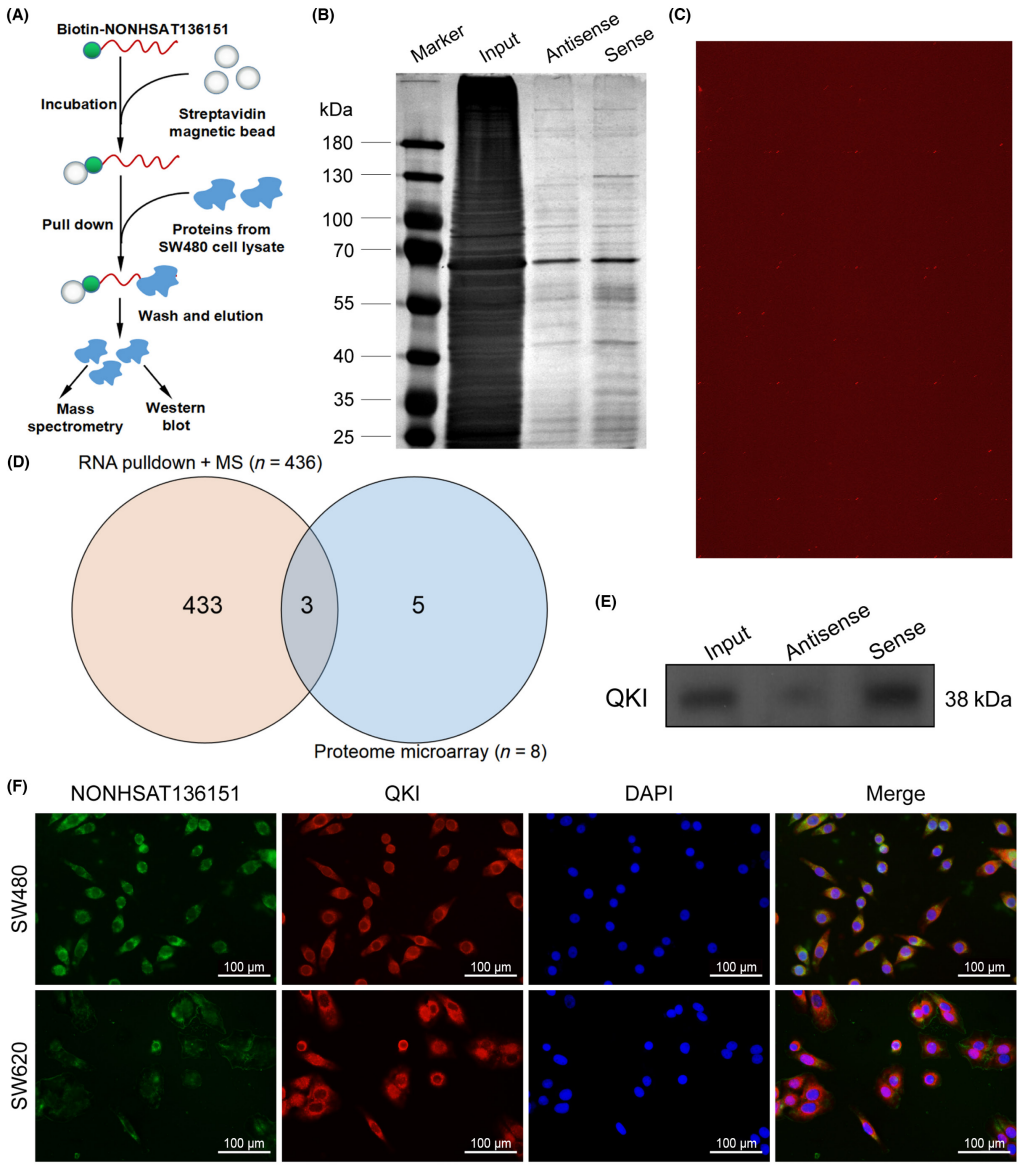
NONHSAT136151 interacted with RBP QKI. (A) Schematic diagram of RNA pull‐down assay for the identification of NONHSAT136151‐associated proteins. (B) Image of sliver staining gel presenting the proteins interacted with NONHSAT136151. (C) Image of proteome microarray displaying NONHSAT136151‐associated proteins. (D) Venn diagram indicated the overlap of NONHSAT136151‐associated proteins detected by RNA pull‐down and proteome microarray. (E) Western blotting was used to confirm the interaction between NONHSAT136151 and QKI after RNA pull‐down assays. (F) IF/FISH assays were performed to indicate the partial cytoplasmic co‐localization of NONHSAT136151 and QKI in SW480 and SW620 cells. DAPI, 4′,6‐diamidino‐2‐phenylindole; FISH, fluorescence in situ hybridization; IF, immunofluorescence; MS, mass spectrometry; RBP, RNA binding protein.

Previous evidence revealed that lncRNAs with cytoplasmic distribution can bind to RBPs to form lncRNA‐RBP complexes, which can regulate cytoplasmic events crucial for maintaining structure and function of cell.^21^, ^22^ Due to its cytoplasmic localization, we hypothesized that NONHSAT136151 may function through such a mechanism (lncRNA‐RBP interaction). Hence, we performed RNA pull‐down assays using biotinylated NONHSAT136151 or a negative control transcript (Figure 5A). The enriched products from SW480 cells were resolved by SDS‐PAGE and stained with silver (Figure 5B). Subsequently, mass spectrometry was performed, and 436 proteins were detected as potential interactive RBPs of NONHSAT136151. In parallel, we performed proteome microarray analysis to screen for NONHSAT136151‐binding proteins (Figure 5C), and eight proteins were identified as candidates. Using overlap analysis of the RNA pull‐down and proteome microarray assays, we identified three putative proteins, QKI, KCNAB1 and HIST1H1B (Figure 5D, Table S4). QKI has been shown to be an RBP with tumour‐suppressant effects in multiple cancers,^23^, ^24^, ^25^, ^26^, ^27^, ^28^, ^29^, ^30^, ^31^, ^32^, ^33^ including colon cancer,^33^ whereas little literature linked KCNAB1 or HIST1H1B with cancer. Therefore, we focused on QKI in the subsequent study and performed western blotting to analyse protein samples from the RNA pull‐down. Results confirmed that QKI was specifically pulled down by NONHSAT136151 but not the antisense RNA (Figure 5E). Additionally, we performed RNA FISH and immunofluorescence (IF) experiments in CRC cells and confirmed that NONHSAT136151 and QKI are partially colocalized in the cytoplasm, indicating that NONHSAT136151 is totally feasible to interact with QKI (Figure 5F). Taken together, these data verified that NONHSAT136151 interacts with QKI in CRC cells.

### 3.6 NONHSAT136151 affected the interaction of QKI with target mRNAs without altering the expression level of QKI

**FIGURE 6:**
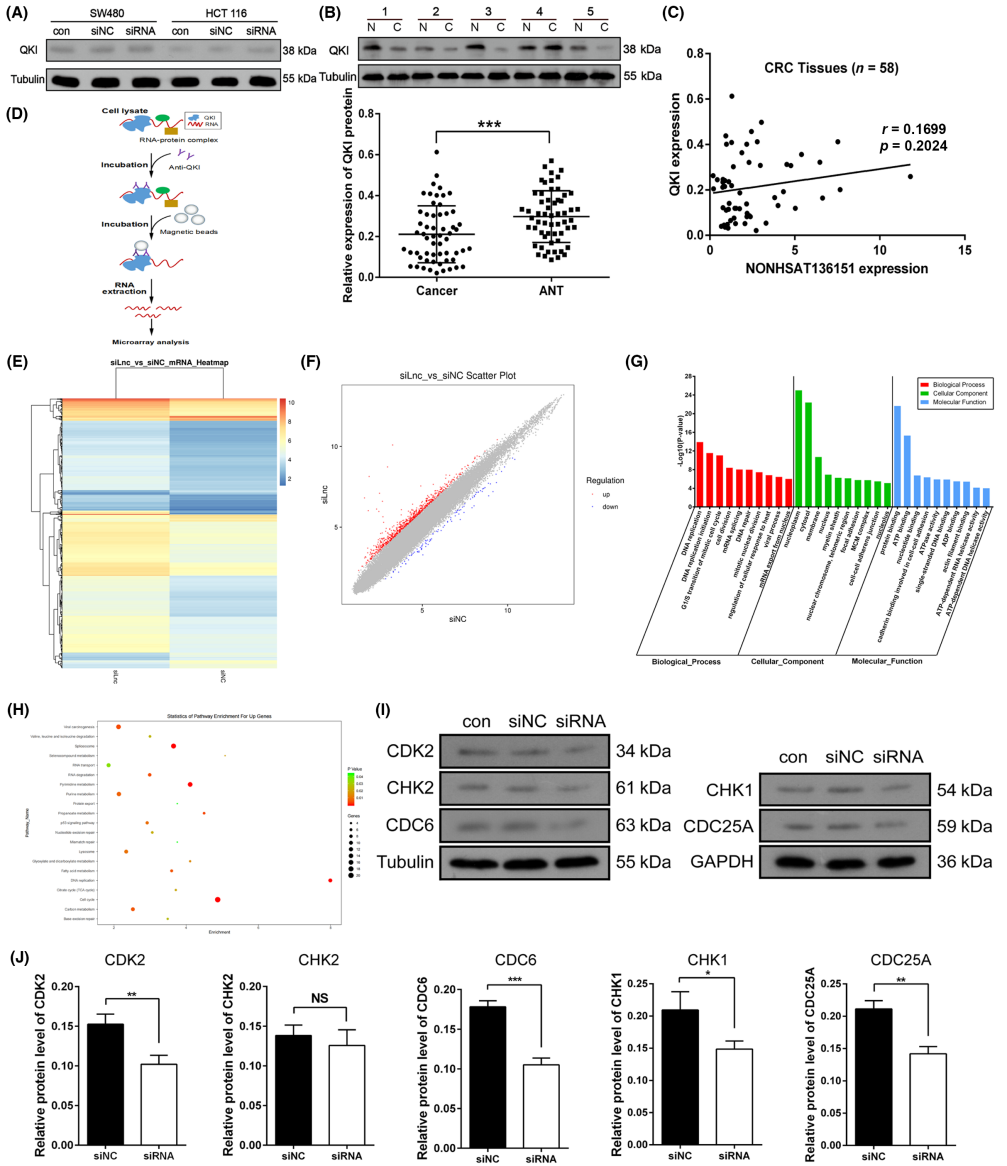
NONHSAT136151 interfered with QKI binding to its target mRNAs without altering QKI expression. (A) Western blotting showed that silencing NONHSAT136151 failed to change the expression of QKI in SW480 and HCT116 cells. (B) QKI expression analysis in CRC tissues and corresponding ANTs using western blot. Upper panel: Representative blots of QKI expression in tissue samples; Lower panel: Differential expression analysis of QKI in paired CRC and ANTs samples (*n* = 58). (C) The correlation analysis showed no significant association between the expression of NONHSAT136151 and QKI protein in CRC tissues (*n* = 58). (D) Schematic diagram of the RIP assays examining the binding of QKI to its target mRNAs in SW480 cells. (E, F) Heatmap and scatter plot represented the differential genes that QKI binding before and after NONHSAT136151 knockdown based on the data of RIP assays. (G) GO function enrichment analysis of DEGs was performed. Top 10 upregulated GO terms of three categories (Biological process, Cellular component and Molecular function) were shown. (H) Bubble diagram of pathways associated with upregulated genes was constructed based on KEGG enrichment analysis. (I, J) Western blot showed the expression changes of 5 key target genes of QKI following NONHSAT136151 knockdown in SW480. *p < 0.05, **p < 0.01, ***p < 0.001. ANT, adjacent noncancerous tissue; CRC, colorectal cancer; DEGs, Differential expressed genes; GO, Gene Ontology; KEGG, Kyoto Encyclopedia of Genes and Genomes; NC, negative control; NS, no significant; RIP, RNA immunoprecipitation.

According to the results above, we surmised that NONHSAT136151 may contribute CRC progression through directly interacting with QKI. First, we hypothesised that NONHSAT136151 regulated expression of the QKI protein in CRC cells. However, expression of QKI by western blotting showed QKI level was not significantly altered after NONHSAT136151 knockdown in SW480 and HCT 116 cells (Figure 6A). Furthermore, QKI expression of 58 CRC samples was examined using western blotting. Consistent with the results of previous studies,^34^, ^35^ a lower level of QKI protein was detected in CRC tissues compared to ANTs (Figure 6B). However, the correlation analysis conducted between NONHSAT136151 and QKI expression did not yield statistically significant results (Figure 6C). These findings indicate that NONHSAT136151 may potentially interact with QKI in a manner that does not exert an influence on its protein expression.

Recent evidence showed that QKI suppressed cancer progresses through interacting with cytoplasmic target mRNAs.^27^, ^31^ Thus, we examined NONHSAT136151's impact on QKI's interaction with mRNA in SW480 cells. NONHSAT136151 knockdown cells were lysed for RNA immunoprecipitation. QKI‐RNA complexes were enriched using QKI IP‐antibodies attached to magnetic beads, and QKI‐binding RNAs were collected after purification (Figure 6D). Subsequently, we analysed the RNA samples via mRNA microarray. The results demonstrated that hundreds of mRNAs co‐precipitated with QKI were altered in RNA abundance after downregulation of NONHSAT136151 in SW480 cells, including 532 upregulated and 72 downregulated mRNAs (Figure 6E,F). Overall, NONHSAT136151 knockdown predominantly enhanced the binding capacity of QKI with its target mRNAs. Furthermore, GO and KECG enrichment analysis were performed to evaluate functional classification of these upregulated mRNAs (Figure 6G,H). Upregulated genes were to a great extent concentrated in the GO terms and KEGG pathways essential to cell growth and maintenance such as DNA replication, cell cycle, cell division, DNA repair, mRNA splicing and pyrimidine/purine metabolism, which were closely related to tumour pathogenesis. Furthermore, we detected expression level of five key genes in DNA replication pathway in SW480 after silencing NONHSAT136151. Previous studies demonstrated the protumor function in CRC of the five genes, CDK2,^36^, ^37^ CHK2,^38^, ^39^ CDC6,^40^, ^41^ CHK1^42^ and CDC25A.^43^ In our study, all five genes expression was reduced to some extent and four genes (CDK2, CDC6, CHK1 and CDC25A) showed the significantly decreased expression (Figure 6I,J), which can partly explain that decreased NONHSAT136151 expression leads to the inhibition of CRC progression.

Collectively, the aforementioned data demonstrate that the interaction between NONHSAT136151 and QKI potentially influences the binding of QKI to its target mRNA, consequently resulting in alterations in the expression of QKI's target genes. This mechanism could potentially underlie the oncogenic activities exerted by NONHSAT136151 in CRC.

### 3.7 FOXP3 regulated the transcription of NONHSAT136151 in CRC cells

**FIGURE 7:**
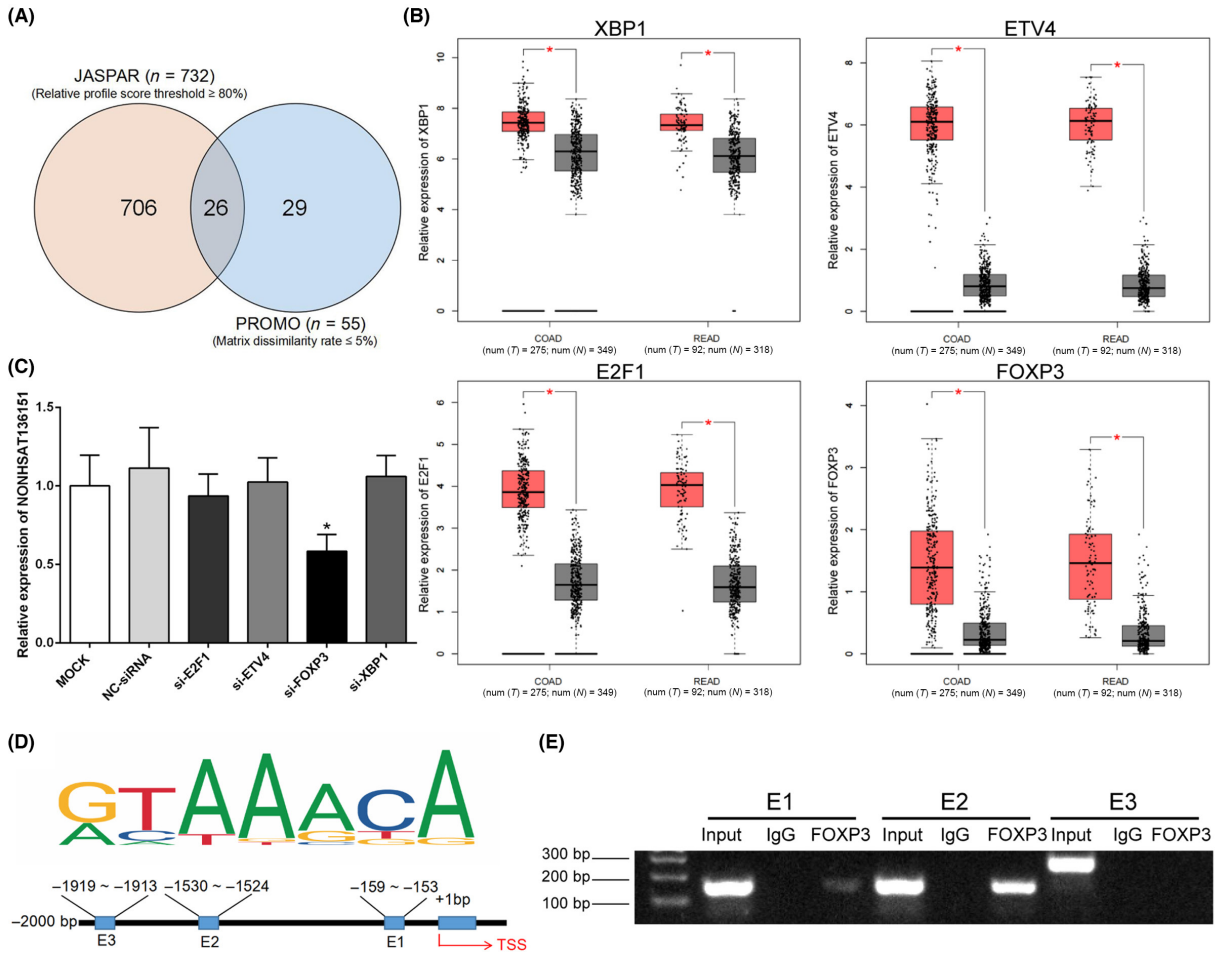
FOXP3 was a potential transcription factor (TF) inducing the expression of NONHSAT136151 in CRC. (A) Venn diagram showed the putative TFs that bind NONHSAT136151 promoter based on JASPAR and PROMO databases. (B) Four upregulated TFs in CRC were screened out using GEPIA from the candidates. (C) RT‐qPCR was used to evaluate the expression of NONHSAT136151 after silencing the four candidate TFs (*n* = 3). (D) The putative FOXP3 binding sites (E1, E2 and E3) in NONHSAT136151 promoter and corresponding binding motif were predicted by JASPAR. (E) ChIP assays were carried out in SW480 cells using antibodies against FOXP3 or IgG. The specific primers were used to detect NONHSAT136151 promoter fragments. *p < 0.05 vs. the NC group. CRC, colorectal cancer; GEPIA, Gene Expression Profiling Interactive Analysis; TF, transcription factor.

Additionally, we explored the upstream molecules that regulate the transcription of NONHSAT136151. First, we retrieved the sequence upstream of NONHSAT136151 transcription start site (TSS) by 2000 base pairs as its theoretical promoter region. Twenty‐six candidate transcription factors were identified by overlapping the JASPAR and PROMO databases (Figure 7A). Using the online tool GEPIA, we subsequently screened out four transcription factors (ETV4, FOXP3, E2F1 and XBP1) which are highly expressed in CRC and closely related to cancer (Figure 7B). Then, we silenced the four transcription factors using siRNAs and analysed NONHSAT136151 expression in SW480. Results indicated that FOXP3 was the only transcription factor that resulted in a significant downregulation of NONHSAT136151 after knockdown (Figure 7C). Subsequently, using JASPAR database, we predicted the potential motifs FOXP3 bind and the three corresponding sites in NONHSAT136151 promoter region with the highest scores (Figure 7D, Table S5). A ChIP assay and PCR were conducted, and the results showed a significant enrichment of FOXP3 on the putative E2 binding site but not the E1 and E3 sites (Figure 7E). According to these data, FOXP3 could be considered as a transcription factor regulating NONHSAT136151 expression of in CRC.

## 4 DISCUSSION

Globally, CRC has a reputation for being one of the most common and lethal cancer types. As evidenced by growing research, lncRNA contributes significantly to cancer biology, including CRC.^44^ Nevertheless, the majority of lncRNAs lack sufficient functional annotation. Our current research has identified a lncRNA, NONHSAT136151, associated with CRC and mainly present in the cytoplasm. Previous evidence suggested that lncRNAs are preferentially localized in the nucleus.^45^ However, recent studies revealed that cytoplasmic lncRNAs are present in greater numbers than previously thought^46^, ^47^ and exhibit higher stability than their nuclear counterparts.^22^, ^47^ The biological function of cytoplasmic lncRNAs may be vastly underestimated. In our study, NONHSAT136151 had a mainly cytoplasmic distribution and high cellular expression and was associated with patients' clinical progress in CRC. NONHSAT136151 knockdown can impair the proliferation, invasion and migration of CRC cells in vitro as well as tumorigenicity in vivo. Therefore, we deem that cytoplasmic NONHSAT136151 is a potential pro‐oncogenic factor of CRC.

It is well known that forming complexes with RBPs is an important mode of lncRNAs function in the nucleus, while cytoplasmic lncRNA‐associated ribonucleoprotein complexes (lncRNPs) can also govern essential events important to cellular structural and functional maintenance.^21^, ^22^ In the current study, we detected the binding proteins of NONHSAT136151 both by RNA pulldown assay and proteome microarray and identified three proteins that interact with NONHSAT136151 (QKI, KCNAB1 and HIST1H1B). There has been a noticeable amount of research relating QKI to cancer pathogenesis, which includes CRC.^23^, ^24^, ^25^, ^26^, ^27^, ^28^, ^29^, ^30^, ^31^, ^32^, ^33^ However, there is little literature to link KCNAB1 or HIST1H1B with cancer. Therefore, our study focused on QKI and did not analyse the other two proteins. Moreover, the co‐localization of NONHSAT136151 and QKI in the cytoplasm further verified that QKI is a specific binding protein of NONHSAT136151. QKI is a classical RBP that belongs to the STAR family and is accepted as a tumour suppressor in CRC as well as many other human cancers. As a multifunctional RBP, QKI predominantly functions by binding to RNA, and increasing evidence has revealed its vital roles in the metabolic processes of multiple RNA species, such as mRNA stabilization,^31^ microRNA processing,^48^ alternative pre‐mRNA splicing^49^ and circular RNA biogenesis.^50^ Nevertheless, there is limited knowledge regarding the significance of the interaction between lncRNA and QKI in the development of cancer. Our research findings suggest that the interaction between NONHSAT136151 and QKI may potentially contribute to a previously unidentified regulatory mechanism associated with CRC.

Previous evidence demonstrated that the process of lncRNA interacting with RBP was often accompanied by a variation in RBP expression and that lncRNAs can modulate the stability of interacted RBP through protein degradation pathways.^16^, ^51^, ^52^ Nevertheless, our study detected no significant change in QKI protein expression in CRC cells after NONHSAT136151 knockdown. Consistently, NONHSAT136151 was not statistically significantly correlated with the level of QKI in CRC tissue. Both results demonstrated that interaction of NONHSAT136151 did not contribute to a significant change in the QKI protein expression. Aside from altering their expression level, lncRNA can also regulate the RBP interaction with their target mRNAs in the cytoplasm after binding to RBPs.^53^, ^54^, ^55^, ^56^ We learned that the QKI protein can function both inside and outside of the nucleus, and modulating the target mRNAs is a prominent way in which it participates in cellular processes in the cytoplasm.^57^ Regarding the target mRNAs of QKI, Galarneau A et al.^58^ revealed that 24% were associated with cell growth and/or maintenance, which was an essential component of cancer pathogenesis. Furthermore, some important mRNAs closely related to cancer such as Fos, Jun, Ras, p53,^58^ β‐catenin^28^ and RASA1,^31^ have been reported as target genes regulated by QKI protein. Thus, it is conceivable that interaction with target mRNAs is highly important to the anti‐oncogenic function of QKI. Our study showed that the abundance of mRNAs enriched by QKI was predominantly enhanced after silencing NONHSAT136151 in CRC cells, indicating that NONHSAT136151 impaired the mRNA binding capacity of QKI. Consistent with a previous study,^58^ the differentially enriched mRNAs identified in our study were largely concentrated in the gene category of cell growth and/or maintenance, including DNA replication, cell cycle, cell division and DNA repair. Furthermore, after NONHSAT136151 knockdown, we detected the expression of several representative genes in DNA replication pathway, which have been reported as cancer‐promoting factors in CRC. Most of these genes showed the decreased level of protein expression, which means that enhancement of the interaction between QKI and its target genes after downregulating NONHSAT136151 can indeed result in the expression alteration of QKI's target genes at the protein level. Based on the aforementioned evidence, it is discernible that QKI primarily exerts its tumour suppressor function by interacting with its target genes. The disruption of QKI‐mRNA interaction by NONHSAT136151 may serve as a significant catalyst in the advancement of CRC.

Like mRNA, regulation of lncRNA transcription is mediated by transcription factors.^59^, ^60^ In our study, transcription factor FOXP3 was found to associate with NONHSAT136151 expression in CRC cells. ChIP further confirmed FOXP3 binding to the promoter region of NOHSAT136151, indicating that FOXP3 might induce the dysregulation of NONHSAT136151 transcription in CRC. FOXP3 is one of members of Forkhead Box family and is well known as a specific marker of regulatory T cells (T_reg_).^61^ As the crucial component of tumour microenvironment, FOXP3^+^ T_reg_ cell infiltration into a tumour can suppress anti‐tumour immunity, thereby inducing immune evasion and promoting tumour progression.^62^ However, previous evidence suggested that FOXP3 can be detected in various cancers and plays dual roles in cancer pathogenesis.^63^, ^64^ FOXP3 plays a protumor role in several cancers^65^, ^66^, ^67^, ^68^ including CRC,^65^ while the antitumor role is observed in other cancers.^69^, ^70^, ^71^ Therefore, to date, the significance of FOXP3 expressed in cancer remains under debate. Our results showed the potential regulation of NONHSAT136151 by FOXP3, suggesting that NONHSAT136151 may be a novel pathway for FOXP3 to be involved in tumour microenvironment regulation. The further study of FOXP3 may be a promising but complex work which requires a significant investment, and it may be possible for our future study to be based on the findings from the current research.

In conclusion, NONHSAT136151 was identified as a potential regulatory lncRNA in CRC pathogenesis in our study. Mechanistically, NONHSAT136151 interacted with the RBP QKI without changing its expression but by interfering with QKI binding to target mRNAs and regulating their expression. Additionally, we partially revealed the regulation of NONHSAT136151 expression by FOXP3, indicating a potential role of NONHSAT136151 in contributing to the tumour microenvironment. Therefore, NONHSAT136151 would be a potential target in CRC treatment.

## AUTHOR CONTRIBUTIONS

**Handong Huang:** Investigation (equal); methodology (equal); writing – original draft (equal); writing – review and editing (equal). **Xiaoxiang Liang:** Investigation (equal); project administration (equal). **Weizheng Wu:** Formal analysis (equal). **Tao Yuan:** Investigation (equal). **Zhengquan Chen:** Project administration (equal). **Lin Wang:** Visualization (equal). **Zhenyu Wu:** Data curation (equal). **Tao Zhang:** Resources (equal). **Kai Yang:** Validation (equal). **Kunming Wen:** Conceptualization (lead); supervision (lead).

## CONFLICT OF INTEREST STATEMENT

The authors confirm that there are no conflicts of interest.

## Supporting information


Tables S1–S5.

## Data Availability

The datasets used and/or analysed during the current study are included in this published article (and in the supplementary files). The datasets of RNA and protein microarrays are available in the following open access repositories: GEO, https://www.ncbi.nlm.nih.gov/geo/ (GEO accession numbers: GSE227471 and GSE227550); Protein Microarray Database, http://www.proteinmicroarray.cn/ (Accession number: PMDA193). Any further data are available from the corresponding author on reasonable request.
