# Supplementary material for: FOXP3‐regulated lncRNA NONHSAT136151 promotes colorectal cancer progression by disrupting QKI interaction with target mRNAs
Source: J Cell Mol Med. 2023 Dec 2;28(2):e18068. doi: 10.1111/jcmm.18068 (PMC10826441; doi:10.1111/jcmm.18068)
Supplement: Supplementary file 1 — Tables S1–S5. [file JCMM-28-e18068-s001.docx]

**Table SI.** **siRNA sequences.**

| **Name** | **Sequence, 5' - 3'** |
| --- | --- |
| si-NON-1 | UAGGCAUGGUUAGCGUGGUCGGAAUTT |
| si-NON-2 | TTCTTACACAATAGCCAGG |
| si-ETV4 | AATTTGAAGGAGACATCAAGC |
| si-FOXP3 | GCATCATCACACAATCACACA |
| si-E2F1 | AAGTCACGCTATGAGACCTCA |
| si-XBP1 | GGAACAGCAAGTGGTAGATTT |
| si-NC | TTCTTACACAATAGCCAGG |

**Table SII. PCR primer sequences.**

| **Name** | **Forward primer (5’-3’)** | **Reverse primer (5’-3’)** |
| --- | --- | --- |
| NONHSAT136151 | GAATAATCTCCCAAACCTCAAT | TGGACAGAAAGGCTACACG |
| DUXAP8 | AGCAGTGGTGGGTTCCAT | ATTCCACAGCAGGGTGACTA |
| RP13-157F18.2 | GCATTGCTCAGTCCCAGAA | GCTGCTTCCAATATCCTCCTG |
| NEAT1 | AGGCAGGGAGAGGTAGAAGG | TGGCATGGACAAGTTGAAGA |
| β-Actin | GGGAAATCGTGCGTGACAT | CAGGCAGCTCGTAGCTCTT |
| GAPDH | CGACCACTTTGTCAAGCTCA | AGGGGTCTACATGGCAACTG |
| E1 binding site | TTTACTATTCCTTTGCACCCTTCA | GTTTGGGAGATTATTCGGACATG |
| E2 binding site | CCCAAATCAGAAGCGTTTAGGTT | GTCTCACAGTGGAGGGAAGGAAT |
| E3 binding site | CCCAAGCGTCGCTGAGTCTTTCT | ATTACAGGGTGGAGGAGCGGAGG |

**Table SIII. Primary and secondary antibodies used in WB.**

| **Antigen** | **WB dilution** | **Antibody name** | **Catalog number** | **Company** |
| --- | --- | --- | --- | --- |
| α-tubulin | 1:4000 | Alpha tubulin rabbit pAb | 11224-1-AP | Proteintech |
| GAPDH | 1:4000 | Anti-GAPDH rabbit pAb | ab9485 | Abcam |
| QKI | 1:1000 | Anti-QKI rabbit pAb | HPA019123 | Sigma-Aldrich |
| ETV4 (Pea3) | 1:1000 | Anti-Pea3 mouse mAb | ab70425 | Abcam |
| FOXP3 | 1:1000 | FOXP3 rabbit pAb | 22228-1-AP | Proteintech |
| E2F1 | 1:1000 | Anti-E2F1 rabbit mAb [1D12] | ab288369 | Abcam |
| XBP1 | 1:1000 | Anti-XBP1 rabbit mAb [EPR4086] | ab109221 | Abcam |
| Rabbit IgG | 1:5000 | Goat anti-rabbit IgG-HRP | ab6721 | Abcam |
| Mouse IgG | 1:10000 | Goat anti-mouse IgG-HRP | ab6789 | Abcam |

**Table SIV. List of proteins after RNA pulldown and proteosome microarray assays.**

| **RNA pulldown** | A2M, ABCF1, ACACA, ACADVL, ACOT7, ACOT9, ACTA1, ACTB, ACTG1, ACTN1, ACTN4, ACTR2, ADAR, ADARB1, ADRM1, AFP, AHCY, AHSA1, AHSG, AIMP1, AIMP2, AK3, ALB, ALDH18A1, ALDH3A2, ALDH9A1, ALDOA, ALDOC, ANP32A, ANXA1, ANXA11, ANXA2, ANXA3, ANXA4, ANXA7, AP2B1, AP3B1, APEH, APEX1, APMAP, APOBEC3B, ARHGDIA, ASS1, ATAD3A, ATIC, ATP1A1, ATP1B1, ATP1B3, ATP2A2, ATP5A1, ATP5B, ATP5C1, ATP5O, BLVRA, BLVRB, BSG, BUB3, C14orf166, CACYBP, CAD, CALR, CAND1, CAP1, CAPG, CAPN1, CAPN2, CAPRIN1, CAPZA2, CAPZB, CCDC177, CCT2, CCT3, CCT4, CCT6A, CCT7, CCT8, CD63, CDC37, CDC2, CDPF1, CFAP74, CFL1, CKAP4, CKAP5, CLIC1, CLPB, CLTC, CMTR1, CNGB3, CNN2, COG3, COPA, COPB1, COPB2, COPG1, CPNE1, CPPED1, CRYZ, CS, CSE1L, CSNK1A1, CTNNB1, CTNND1, CTSB, CTSD, CUL4B, CYFIP1, CYP2S1, DARS, DCD, DCXR, DDB1, DDOST, DDX1, DDX17, DDX21, DDX39B, DDX41, DDX46, DDX5, DDX58, DHX15, DHX30, DHX9, DICER1, DKC1, DLST, DNAJA1, DPYSL3, DRG1, DSG1, DSP, DYNC1H1, DYNC1LI2, ECH1, EEF1A1P5, EEF1D, EEF1G, EEF2, EHD1, EHD4, EIF2AK2, EIF2S1, EIF2S2, EIF2S3, EIF3F, EIF4A1, EIF4A3, EIF4G1, EIF6, ELAVL1, ENO1, EPHA2, EPPK1, EPRS, ERLIN1, ERO1L, ETFA, EZR, FAM98A, FARSA, FASN, FBL, FHL2, FKBP3, FLII, FLNA, FLNB, FUS, FXR1, G6PD, GALC, GAPDH, GART, GCK, GDI2, GFPT1, GLO1, GLUD1, GMPS, GNAS, GNB2L1, GNL3, GOT2, GPI, GSN, GSTO1, GSTP1, GTF2I, H2AFV, HADH, HADHA, HADHB, HARS, HBS1L, HDLBP, HIST1H1B, HIST1H1D, HLA-A, HMGB1, HNRNPA0, HNRNPH1, HNRNPK, HNRNPL, HNRNPM, HNRNPR, HNRNPU, HNRNPUL1, HNRNPUL2, HS2ST1, HSD17B4, HSPA6, IGF2BP2, IGF2BP3, IGHA2, IGHG1, IGKV2-40, IL18, ILF2, ILF3, IMMT, IMPDH2, IPO4, IPO5, IPO7, IQGAP1, ITGA2, ITGA3, ITIH2, JUP, KARS, KCNAB1, KHSRP, KPNA2, KPNB1, KTN1, LAMC1, LAMP1, LARS, LDHA, LDHB, LGALS3, LMNA, LMNB1, LONP1, LYZ, MARS, MATR3, MBLL, MCM3, MCM5, MDH1, METAP2, MSN, MT-ATP6, MYH14, MYH9, MYO1C, MYO1D, MYOF, N4BP2L2, NACA, NAP1L1, NAPA, NARS, NCKAP1, NCL, NNMT, NONO, NOP56, NOP58, NPEPPS, NPM1, NPM3, NQO1, NSUN2, NUP98, OASL, OAT, P4HB, PA2G4, PABPC1, PAFAH1B3, PARP1, PC, PCBP2, PCCA, PCCB, PCNA, PDCD6IP, PDHA1, PDHB, PDIA3, PDIA3, PDIA4, PDIA5, PKP2, PLEC, PLIN3, PLS3, PLXNA3, PNKP, PNP, PNPT1, POR, PPA1, PPIB, PPP1CC, PPP2CB, PPP2R1A, PPT1, PRDX1, PRDX4, PRDX6, PRKDC, PRMT1, PRPF19, PRPF40A, PRPF40A, PRPF6, PRSS3, PSAT1, PSMA1, PSMA3, PSMA4, PSMA6, PSMA7, PSMB4, PSMB5, PSMC1, PSMC3, PSMC4, PSMC6, PSMD11, PSMD14, PSMD2, PSMD3, PSMD8, PTBP1, PYGB, QARS, QKI, RAB1B, RAB21, RAB2A, RAB5C, RAB6B, RAB7A, RAE1, RALY, RAN, RANBP1, RARS, RBBP4, RHOA, RHOC, RNH1, RPA2, RPL10, RPL10A, RPL11, RPL13, RPL14, RPL17, RPL18, RPL19, RPN2, RPS5, RTCB, SAR1A, SERPINB1, SERPINB6, SF3B1, SFPQ, SFXN3, SHMT2, SHROOM3, SLC1A5, SLC25A1, SLC25A11, SLC25A13, SLC4A5, SNRPA1, SNX1, SORD, SRP54, SRP68, SRSF9, SSB, SSR4, STAM2, STAU1, STAU2, STRBP, STT3B, SYPL1, TAGLN, TAGLN2, TAOK3, TARBP2, TARDBP, TARS, TCERG1, TECR, THOC2, TM9SF3, TMED10, TNIK, TOMM22, TOMM40, TOP1, TOR1AIP1, TPM2, TPM4, TPR, TPTE2, TRAM1, TRAP1, TRIM25, TROVE2, TTLL11, TUBA1A, U2AF2, UBA1, UBA2, UQCRC1, VARS, VDAC3, VTA1, WDR77, XPO1, XPO7, YARS, ZBTB8OS, ZC3H15, ZFR |
| --- | --- |
| **Proteosome microarray** | IFT22, KCNAB1, QKI, E2F2, YAF2, HIST1H1B, CRYBB1, SF3B3 |

Note: The overlapped proteins are in red font.

**Table SV. Promotor sequences used to design the E1, E2 and E3 binding site primers (binding site sequences have been marked in red font).**

| **Binding site** | **Sequence** |
| --- | --- |
| E1 | CTAATCACCCTTACGCCGCTCAACGCCAATATCTCATCCCACAGCATGCTTTGAAAGGATTAAAGCCTGTTATCACTCGCCTGCTACAGCATGGGCTTTTAAAACCTATAAACTCTCCTTACCATTCCCCCATTTTACCTGTCCTAAAACCAGACAAGCCTTACAAGTTAGTTCAGAATCTGCGCCTTATCAACCAAATGTTTTGCCTATCCACCCCGTGGTGCCAAACCCATATACTCTTCTATCCTCAATACCTCCCTCTACTACCCATTATTCTGTTCTGGATCTCAAACATGCTTTCTTTACTATTCCTTTGCACCCTTCATCCCAGCCTCCCTTTGCTTTCACTTAGACTGACCCTGACACCCATTAGGCTCAGCAAATTACCTGGGCTGTACTGCCGCAAGGCTTCACAGACAGCCCCCATTACTTCAGTCAAGCCCAAATTTCATCCTCATCTGTTACCTATCTGGGCATAATTCTCATAAAAACACAGGTGCTCTCCCTGCTGATCATGTCCGAATAATCTCCCAAACCTCAATCCCTTACAAAACAACAACTCCTTTCCTTCCTAGGCATGGTTAGCGTGGTCGGAATTCTTACACAA |
| E2 | TCTTCCTTCTTTCCCTCCCGCCTGTCCCCTCAGTCCCAACCCCAAGCGTCGCTGAGTCTTTCTAATCTTCCTTTTCTACAGACCCATCTGACCTCTCCCCTCCTCGCCAGGCCAAGCTAGGTCCCAATTCTTCCTCAGCCTCCGCTCCTCCACCCTGTAATCTTTTTATCGCCTCCCCTCCTCACACCTGGTCCGGCTTACAGTTTCGTTCAGTGACTAGCCCTCCCCCACCTGCCCAGCAATTTACTCTTAAAAAGGTGGCTGGAGCCAAAGGCATAGTCAAGGTTAATGCTCCTTTTTCTTTATCCCAAATCAGAAGCGTTTAGGTTCTTTTTCATCAAATATAAAAACCCAGCCCAGTTCATGGCTCATTCGGCAGCAACCCTGAGACGCTTTACAGCCCTAGACCCTAAAAGGTCAAAAGGCCATCTTATTCTCAATATACATTTTATTACCCAATCTGCTCCCGACATTAAATAAAACTCCAAAAATTAGAATCTGGCCCTCAAACCCCACAACAGGACTTAATTAACCTCACCTTCAAGGTGTACAATAATAAAAAAAAAAGTTGCAATTCCTTCCCTCCACTGTGAGACAAACCCCAGCC |
| E3 | TTTTCTGGAGGGCAAGAACCCCCAACCCCTTCTCCGTGTCTCTACTCTTTTCTCTGGCCTTGCCTCCTTCACTATGGGCAAGCTTCCACCTTCCATTCCTCCTTCTTCTCCCTTAGCCTATATTCTTAAGAACTTAAAACCTCTTCAACTCTCACCTGACCTAAAATCTAAGCATCTTATTTTCTTCTGCAATGCCGCTTGAGGCCAATACAAACTCGACAGTAGTTCCAAATAGCCGGAAAATGGCACTTTCAATTTTTCCATCCTACAAGATCTAAATAATTCTTGTCGTAAAATGGGCAAATATGGTCTGAGGTGCCTGACGTCCAGGCATTCTTTTACACATCAGTCCCTTCCTAGTCTCTGTGCCCAGTGCAACTCGTCCCAAATCTTCCTTCTTTCCCTCCCGCCTGTCCCCTCAGTCCCAACCCCAAGCGTCGCTGAGTCTTTCTAATCTTCCTTTTCTACAGACCCATCTGACCTCTCCCCTCCTCGCCAGGCCAAGCTAGGTCCCAATTCTTCCTCAGCCTCCGCTCCTCCACCCTGTAATCTTTTTATCGCCTCCCCTCCTCACACCTGGTCCGGCTTACAGTTTCGTTCAGTGACT |
